# Beyond osteogenesis: an *in vitro* comparison of the potentials of six bone morphogenetic proteins

**DOI:** 10.3389/fphar.2013.00125

**Published:** 2013-10-01

**Authors:** Jessica C. Rivera, Cassandra A. Strohbach, Joseph C. Wenke, Christopher R. Rathbone

**Affiliations:** Extremity Trauma and Regenerative Medicine, US Army Institute of Surgical Research, JBSAFort Sam Houston, TX, USA

**Keywords:** bone morphogenetic protein, osteoblast, bone marrow stromal cell, osteogenesis, angiogenesis, chemotaxis

## Abstract

Bone morphogenetic proteins (BMPs) other than the clinically available BMP-2 and BMP-7 may be useful for improving fracture healing through both increasing osteogenesis and creating a favorable healing environment by altering cytokine release by endogenous cells. Given the spectrum of potential applications for BMPs, the objective of this study was to evaluate various BMPs under a variety of conditions to provide further insight into their therapeutic capabilities. The alkaline phosphatase (ALP) activity of both C_2_C_12_ and human adipose-derived stem cells (hASCs) was measured after exposure of increasing doses of recombinant human BMP-2, -4, -5, -6, -7, or -9 for 3 and 7 days. BMPs-2, -4, -5, -6, -7, and -9 were compared in terms of their ability to affect the release of stromal derived factor-1 (SDF-1), vascular endothelial growth factor (VEGF), and basic fibroblast growth factor (b-FGF) from human bone marrow stromal cells (hBMSCs). Gene expression of ALP, osteocalcin, SDF-1, VEGF, and b-FGF following shRNA-mediated knockdown of BMP-2 and BMP-6 in hBMSCs or human osteoblasts under osteogenic differentiation conditions was also evaluated. Collectively, BMPs-6 and -9 produced the greatest osteogenic differentiation of C_2_C_12_ and hASCs as determined by ALP. The hBMSC secretion of SDF-1 was most affected by BMP-5, VEGF by BMP-4, and b-FGF by BMP-2. The knockdown of BMP-2 in BMSCs had no effect on any of the genes measured whereas BMP-6 knockdown in hBMSCs caused a significant increase in VEGF gene expression. BMP-2 and BMP-6 knockdown in human osteoblasts caused significant increases in VEGF gene expression and trends toward decreases in osteocalcin expression. These findings support efforts to study other BMPs as potential bone graft supplements, and to consider combined BMP delivery for promotion of multiple aspects of fracture healing.

## Introduction

In 1965, Marshall R Urist published a landmark study on a morphogenetic matrix that affected differentiation of cartilage and bone tissues (Urist, 1965). He continued experimentation with decalcified bone material from which leached a diffusible bone morphogenetic property, and later identified this morphogenetic property as protein though radioactive isotope labeling (Urist, 1970; Urist and Strates, 1971; Nogami et al., 1977). Wozney et al. (1988) later identified these proteins as members of the transforming growth factor (TGFβ) family of growth factors. Of the greater than twenty types of bone morphogenetic proteins (BMPs) that have been identified, two recombinant human BMPs, BMP-2 and BMP-7, are available for limited clinical use. The BMPs vary widely in their genetic profile, protein structure, and effector pathways. Bone regeneration is a complex process involving multiple spatial and temporal interactions among tissues, cells, and growth factors; therefore, the distinct roles BMPs have during bone healing should be taken into account when designing a biological and/or tissue engineering-based therapy.

To date the majority of studies comparing BMPs have focused on the direct osteogenic effects of the different BMPs utilizing cells with relatively robust osteogenic potential, [e.g., osteoblasts, C_2_C_12_ cells, and bone marrow-derived mesenchymal stem cells (BMSCs)]. Comprehensive studies, some using adenoviral approaches and a large number of recombinant BMPs, have demonstrated that BMPs-4, -6, and -9 in addition to the clinically available BMP-2 and -7 have significant osteogenic potential *in vitro* and/or *in vivo* providing enthusiasm for further exploration (Cheng et al., 2003; Li et al., 2003; Kang et al., 2004; Luu et al., 2007; Kang et al., 2009). More recently, the notion that stem and progenitor cells, including mesenchymal stem cells (MSCs), contribute to bone healing through their ability to influence regenerative processes including angiogenesis and chemotaxis has been expanding and is beginning to be considered to play an essential role during tissue regeneration (Guo et al., 2006; Geiger et al., 2007; Marsell and Einhorn, 2011; Shinohara et al., 2011). To gain a comprehensive understanding of the role of the various BMPs on overall tissue regeneration, their effects on the secretion of factors by MSCs important for other important processes during healing, (i.e., angiogenesis and cell migration) should be considered in addition to their osteogenic potential.

Tissue engineering strategies for improved fracture healing have been expanding to include the use of MSCs derived from adipose tissue (adipose-derived stem cells, ASCs) due to the relative tissue abundance and potential to provide a large number of MSCs (Kim et al., 2012). Since differences exist among BMPs on cells well-documented to possess a high osteogenic capacity, a difference also likely exists among them for ASCs as well and may prove to be useful in maximizing their therapeutic potential. Therefore, in the current study the effects of various BMPs with regards to their osteogenic potential for ASCs, and their ability to influence the secretion of regenerative factors of MSCs were determined. Increasing the levels of BMPs for the purpose of improving fracture healing is indeed valuable to maximize their potential. However, relatively less is known about the consequences subsequent to decreases in the levels of BMPs. To this end, to begin to gain insight into the effects of decreased endogenous levels of BMPs-2 and 6, shRNA was used to decrease their mRNA levels in both BMSCs and osteoblasts and resultant changes in genes important for overall fracture healing measured.

## Materials and methods

### Alkaline phosphatase assay (ALP)

Human adipose-derived stem cells (hASCs) (PromoCell USA, Heidelberg, Germany) were cultured in alpha-MEM (Invitrogen™, Carlsbad, CA) containing 10% fetal bovine serum (FBS) (Thermo Scientific, Waltham, MA) and 1% Antibiotic-Antimycotic (Invitrogen™, Carlsbad, CA). C_2_C_12_ mouse myoblasts (ATCC®, Manassas, VA) were cultured in Dulbecco's Modified Eagle's Medium (DMEM, ATCC®, Manassas, VA) containing 10% FBS and 1% Antibiotic-Antimycotic. C_2_C_12_ cells (*n* = 5–6 wells/dose/BMP) or ASCs (*n* = 3 wells/dose/BMP) were seeded at 2 × 10^4^ cells/cm^2^ on 24-well tissue culture-treated plates overnight. The next day, BMP-2,- 4,-5, -6, -7, or -9 (all from R&D Systems®, Minneapolis, MN) were added to achieve the desired concentration. Three or 7 days later, cells were washed twice with phosphate buffered saline (PBS) and whole cell extracts were obtained with the addition of 200 ul of CelLytic™ M lysis buffer (Sigma-Aldrich®, St. Louis, MO) according to the manufacturer's recommendation. For the 7 days treatment groups, after 3 days of BMP treatment on the C_2_C_12_ cells and ASCs the media was changed to their respective growth media without BMPs for an additional 4 days, after which the cells were lysed for alkaline phosphatase (ALP) activity. ALP was determined by incubating lysates with the p-nitro phenyl phosphate phosphatase (p-NPP) for 30 min (AnaSpec, Inc., Fremont, CA), and the absorbance read at 405 nm using a Spectra Max M2 plate reader and SoftMax Pro 4.7.1 software. ALP readings were normalized to protein achieved with the Bio-Rad Bradford Protein assay (Bio-Rad Laboratories, Inc, Richmond, VA).

### Enzyme linked immunosorbent assay (ELISA)

Human bone marrow stromal cells (hBMSCs) (STEMCELL Technologies, Vancouver, Canada) were cultured in alpha-MEM containing 10% FBS and 1% Antibiotic-Antimycotic. hBMSCs were seeded at 2 × 10^4^ cells/cm^2^ on 24-well tissue culture-treated plates overnight. The next day, BMP-2,- 4,-5, -6, -7, or -9 were added to achieve the desired concentration. Supernatants were collected from hBMSCs treated with BMPs (*n* = 3 wells/dose/BMP) 3 days later. Stromal-derived factor 1 (SDF-1; R&D Systems®, Minneapolis, MN), Vascular endothelial growth factor (VEGF; R&D Systems®, Minneapolis, MN), and basic fibroblast growth factor (b-FGF; Invitrogen™, Carlsbad, CA) enzyme linked immunosorbent assay (ELISA) assays were completed as per the respective manufacturer's recommendation. ELISA results from each well were normalized to its respective cell number as determined by the CyQUANT® assay (Invitrogen™, Carlsbad, CA) where cell lysates were incubated in fluorescent dye and the cell lysis buffer from the CyQUANT® DNA assay kit for 10 min. The fluorescent intensity was determined on a SpectraMax M2 microplate reader with software SoftMax Pro 4.7.1 with excitation at 480 nm and emission at 520 nm and adjusted to a standard curve.

### ShRNA transduction

hBMSCs (STEMCELL Technologies, Vancouver, Canada) were seeded (2–3 wells/condition) at 2 × 10^4^ cells/cm^2^ on 24-well tissue culture-treated plates overnight. Twenty-four hours after seeding, cells were treated with osteogenic induction media consisting of alpha-MEM containing 10% FBS, 2 mM L-glutamine, absorbic acid (50 ug/ml), β -glycerophosphate (5 mM), and dexamethasone (10 nM), and 0.001% antibiotic-antimycotic. Twenty-four hours later cells were infected at a multiplicity of infection of 5 with control, BMP-2, or BMP-6 shRNA lentivral particles in osteogenic media containing polybrene (all from Santa Cruz Biotechnology, Santa Cruz, CA). Twenty-four and forty-eight hours later the media was replaced with osteogenic induction media and osteogenic induction media containing puromycin, respectively. On the sixth day (after three total days of shRNA knockdown) cells were lysed for mRNA analyses as described below. Knockdown of BMP-7 mRNA and BMP-9 mRNA using shRNA lentiviral particles was attempted, however, under the real-time polymerase chain reaction (PCR) conditions we utilized BMP-7 and BMP-9 mRNA levels were too low to reproducibly measure.

### Real-time quantitative polymerase chain reaction (PCR)

Total RNA was isolated from cells using RNeasy® Mini Kit (Qiagen, Valencia, CA) according to the manufacturer's instructions. Contaminating DNA was removed with DNAse I treatment during the purification process. cDNA was synthesized using the SuperScript® III First-Strand Synthesis SuperMix (Invitrogen, Carlsbad, CA) and 100 ng of total RNA in a 20 μl volume making a cDNA concentration of 5 ng/μl. Real-time PCR was performed with 2 μl of cDNA, 1 μl RT^2^ qPCR Primer Assay (10 μM stock) and 12.5 μl of SYBR Green (QuantiTect SYBR Green PCR Kit, Qiagen) in an iQ5 PCR Thermal Cycler (Bio-Rad Laboratories, Inc, Richmond, VA). PCR amplification conditions were 95°C for 10 min, 40 cycles of 15 s at 95°C and 1 min at 60°C. Primer sets used to create amplicons from mRNA were all pre-designed and validated by SABiosciences (Qiagen, Valencia, CA). Relative mRNA abundances were quantified as Ct (cycle threshold value) relative to the Ct of GAPDH, a housekeeping gene, based on the assumption that cell GAPDH mRNA levels are constant and RT and PCR reaction efficiencies are constant. Expression levels are shown as fold or percentage increases or decreases in mRNA levels as calculated by the 2^−ΔΔCt^ method (Livak and Schmittgen, 2001).

### Statistical analysis

Comparisons among BMP treatments were made using an analysis of variance (ANOVA) with Tukey–Kramer *post-hoc* analyses where appropriate. Significance for real-time PCR data was evaluated by the Student's two tailed *t*-test. The data are shown as mean ± SEM relative to control. Statistical significance was set at *p* ≤ 0.05.

## Results

### Alkaline phosphatase assay (ALP)

**Figure 1:**
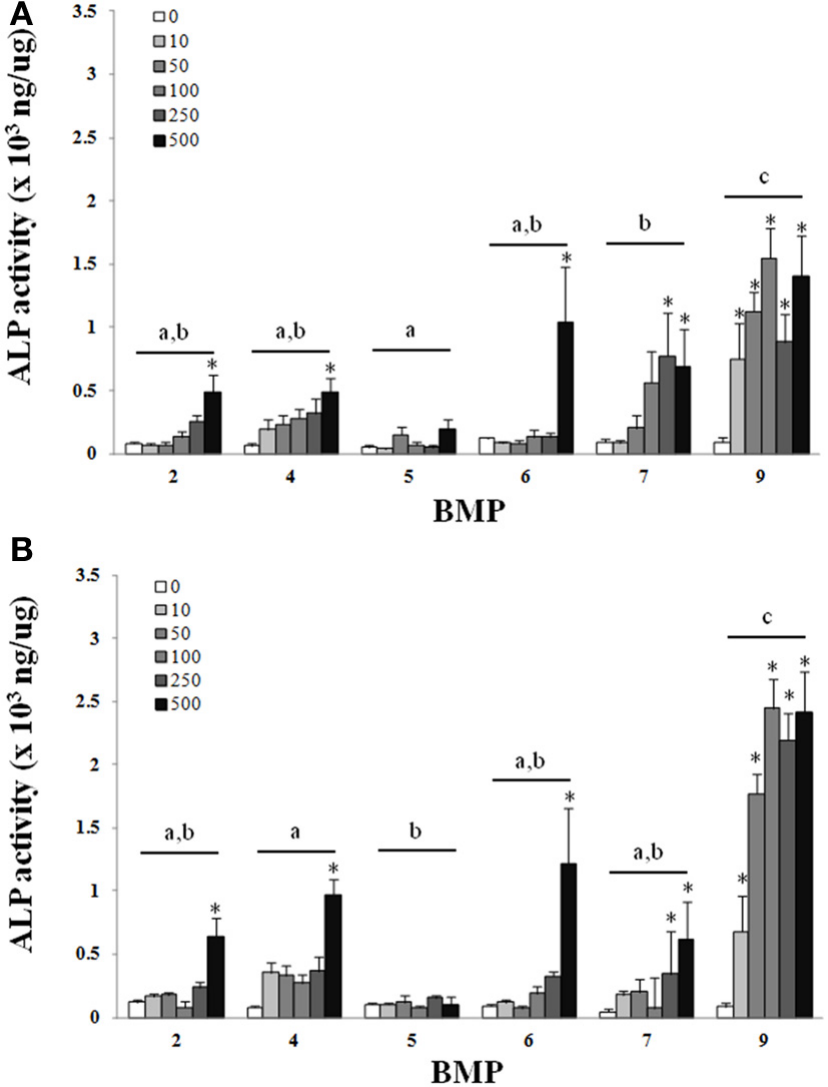
**C_2_C_12_cells were treated with BMP-2,- 4,-5, -6, -7, or -9 for 3 days and lysed on day 3 (A) or 7 (B) after the initiation of treatment.** Error bars are ± SEM. Different letters signify a difference among groups, *p* ≤ 0.05. ^*^Significantly different control (0 ng/ml BMP) within group, *p* ≤ 0.05.

**Figure 2:**
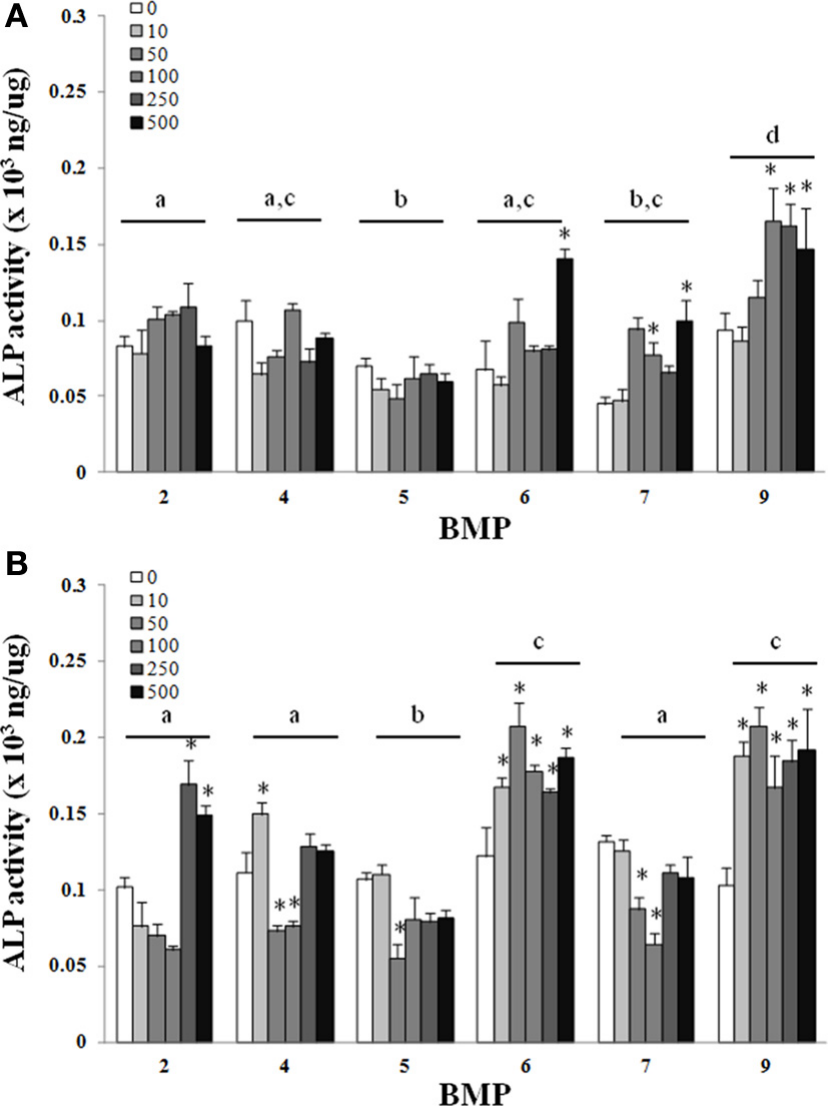
**Adipose-derived stem cells (ASCs) were treated with BMP-2,- 4,-5, -6, -7, or -9 for 3 days and lysed on day 3 (A) or 7 (B) after the initiation of treatment.** Error bars are ± SEM. Different letters signify a difference among groups, *p* ≤ 0.05. ^*^Significantly different control (0 ng/ml BMP) within group, *p* ≤ 0.05.

There was a main effect of BMP type on ALP for both the C_2_C_12_ cells (Figure 1) and hASCs (Figure 2) after treatment with BMP-2,- 4,-5, -6, -7, or -9 when lysed on day 3 (Figures 1A, 2A), or exposed to 3 days of treatment followed by 4 days without any BMP (day 7; Figures 1B, 2B) 7 days after the initiation of treatment. For the C_2_C_12_ cells, BMP-9 was the most effective at increasing ALP on both days 3 and 7 where it significantly increased ALP as compared to all of the other BMPs, and ALP was increased relative to control (0 ng/ml) at all doses tested (*p* ≤ 0.05). BMPs-4, -6, and -7 increased ALP compared to control (0 ng/ml) at doses ≥250 ng/ml on both days 3 and 7 (*p* ≤ 0.05). For the hASCs, BMP-9 was the most effective at increasing ALP on day 3 where it significantly increased ALP as compared to all of the other BMPs and was greater than control (0 ng/ml) at doses ≥100 ng/ml (*p* ≤ 0.05). Similar to that observed with the C_2_C_12_ cells, BMPs-6 and -7 increased ALP as compared to control at 500 ng/ml. On day 7, BMPs-6 and -9 were similar and significantly increased ALP as compared to all other BMPs, and ALP was increased relative to control (0 ng/ml) at all doses tested (*p* ≤ 0.05).

### ELISA

**Figure 3:**
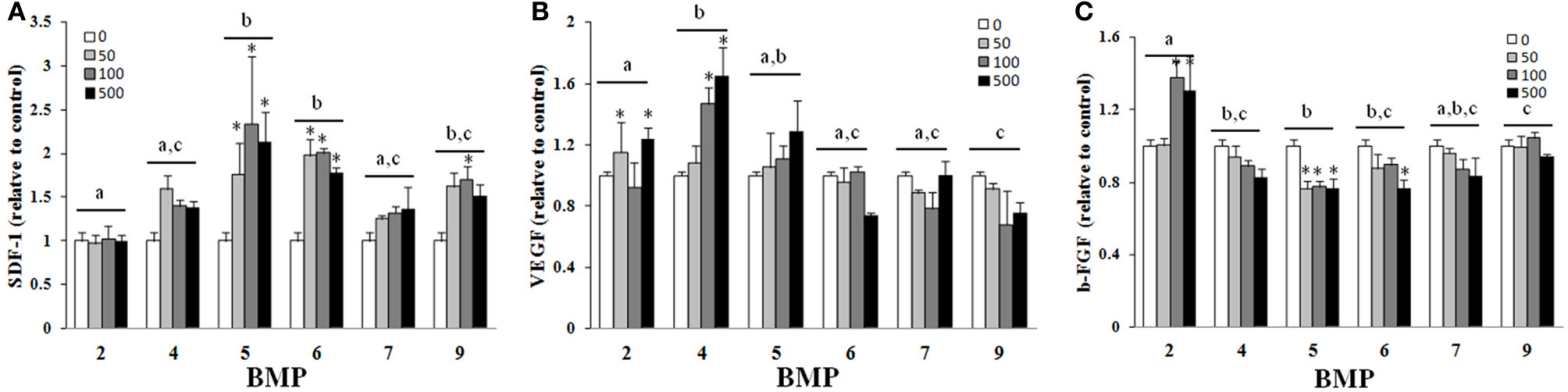
**Effect of different BMPs on SDF-1 (A), VEGF (B), and b-FGF (C).** BMSCs were treated with BMP-2,- 4,-5, -6, -7, or -9 for 3 days, and the supernatant collected for ELISA on the third day. The bar graph represents the mean ELISA value normalized to cell number and is presented as relative to control. Different letters signify a difference among groups, *p* ≤ 0.05. Error bars are ± SEM. Different letters signify a difference among groups, *p* ≤ 0.05. ^*^Significantly different control (0 ng/ml BMP) within group, *p* ≤ 0.05.

There was a main effect of BMP type on SDF-1 (**A**), VEGF (**B**), and b-FGF (**C**) when BMPs were added to hBMSCs for 3 days (Figure 3). BMPs-5, -6, -and -9 were similar and statistically greater than BMPs-2, -4, and 7 with regards to SDF-1 secretion, with doses ≥50 ng/ml and 100 ng/ml greater than control (0 ng/ml) for BMPs-2 and -4, -9, respectively (Figure 3A). BMPs-4 and -5 were similar and statistically greater than all other BMPs with regards to VEGF secretion (Figure 3B). BMP-2 appeared to be most effective in its ability to increase b-FGF secretion as it was significantly greater than all other BMPs, and significantly greater than its own control at both 100 and 500 ng/ml (*p* ≤ 0.05; Figure 3C).

### Real-time PCR

**Figure 4:**
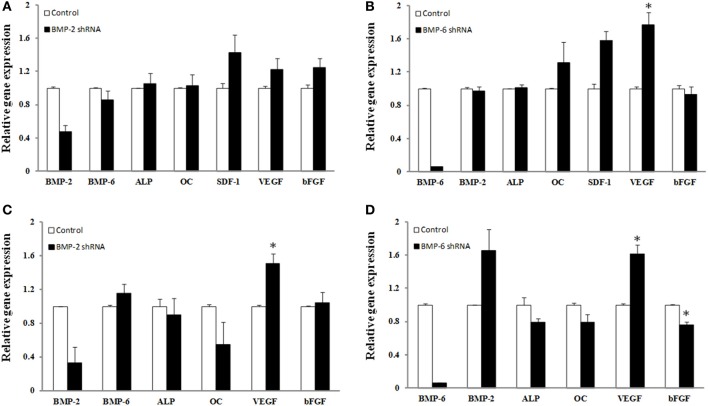
**Effect of shRNA mediated knockdown of BMP-2 (A,C) and BMP-6 (B,D).** BMSCs **(A,B)** or osteoblasts **(C,D)** were infected with control, shRNA BMP-2 **(A,C)** or shRNA BMP-6 **(B,D)** lentiviral particles in osteogenic induction media. After 3 days of knockdown gene expression for BMP-2, BMP-6, ALP, osteocalcin (OC), SDF-1, VEGF, and b-FGF was determined. Error bars are ± SEM. ^*^Significantly different control, *p* ≤ 0.05.

The average knockdown for hBMSCs for BMP-2 and BMP-6 was 52 ± 8 and 94 ± 0.2%, respectively (*p* ≤ 0.05) (Figures 4A,B). Whereas the knockdown of endogenous BMP-2 mRNA in human BMSCs resulted in no significant changes in the mRNA expression levels of any of the genes measured, there were trends toward increased mRNA expression of VEGF, bFGF, and SDF-1. BMP-6 mRNA knockdown resulted in a significant increase in VEGF (78% increase) and trends toward increases in SDF and osteocalcin mRNA.

There was a considerable amount of variability with the BMP-2 lentiviral transduction of osteoblasts where the decrease in BMP-2 gene expression ranged from 34 to 99% (Figure 4C). Nonetheless, lentiviral transduction of shRNA for BMP-2 caused a significant change in VEGF gene expression (51% increase). Although statistical significance was not reached, presumably due to the low sample number, an interesting observation regarding osteocalcin expression was that the individual samples with the highest knockdown of BMP-2 mRNA also had the greatest decrease in osteocalcin expression. More specifically, decreases in BMP-2 of 34, 67, and 99% resulted in 2, 41, and 92% decreases in osteocalcin expression, respectively. BMP-6 knockdown was more consistent as the percent decrease in BMP-6 gene expression ranged from 93–95%. As a result, significant changes in VEGF (61% increase) and bFGF (24% decrease) were observed (*p* ≤ 0.05). Also there was a strong trend toward knockdown of BMP-6 leading to an increase in BMP-2 mRNA in osteoblasts. Although not significant, 21% decreases in both ALP and osteocalcin gene expression, and a 66% increase in BMP-2 was measured in the presence of BMP-6 mRNA knockdown (*p* > 0.05) (Figure 4D).

## Discussion

The primary objective of this study was to gain a more comprehensive understanding of the therapeutic benefits of six selected BMPs. While BMPs-2 and -7 have shown promise in a series of clinical trials both in spine and in orthopedic trauma, it is unclear whether or not these are the most effective BMPs for promotion of fracture healing. To this end, recombinant BMPs were compared with regards to their effects on osteogenesis using C_2_C_12_ cells, a model cell line with differentiation potential, and ASCs which are currently being explored for a variety of musculoskeletal applications. To assess and compare the ability of the BMPs to influence the secretory capacity of hBMSCs, growth factors produced by hBMSCs that are important for bone healing (SDF-1, VEGF, and b-FGF) were also evaluated. A final objective was to gain insight into the role of endogenous BMPs which was achieved by decreasing their levels using shRNA.

Despite differences in the dosage, method of growth factor manipulation, and duration of treatment our findings were in agreement with previous studies where the BMPs-2, -4, -6, -7, and -9 produced significant increases in the ALP activity of C_2_C_12_ cells during treatment as compared to untreated controls (Li et al., 2003; Luu et al., 2007). In the current study, BMP-9 was the most effective at increasing ALP when compared directly to all other BMPs. Previously, when compared to BMP-2, 4, and 7, BMP-6 performed the best with regards to the osteogenic differentiation of human MSCs (Friedman et al., 2006). Although their study did not include BMP-9, our results are in agreement with the potential for BMP-6 for osteogenic differentiation, and are in close agreement with others where the adenoviral over-expression of BMPs-6 and -9 were exceptional when using C_2_C_12_ cells (Luu et al., 2007). It is worth emphasizing that the 7 day time point in the current study included the culturing of cells in the presence of BMPs for 3 days, followed by the removal of the BMPs and an additional 4 days of culture with no BMP treatment. Under these circumstances the trend remained consistent between days 3 and 7 and there was an apparent increase in ALP activity between 3 and 7 days despite removal of the BMP, an observation that was the most obvious with BMP-9 (Figures 1, 2). A logical speculation is that bolus of BMPs during the first 3 days was sufficient to initiate osteogenesis that was maintained during the remaining 4 days of culture. For many tissue engineering applications a sought after objective is the controlled and often prolonged release of growth factors *in vivo* (Lee et al., 2011). Conversely, *in vivo* studies have demonstrated the importance of a bolus release of BMPs for bone repair (Li et al., 2009). In light of these concepts, BMP-9 seems to be particularly effective. The treatment with BMP-9 for 3 days, followed by its removal for 4 days, was effective and appears to be particularly suitable for the initiation and maintenance of osteogenesis with a large therapeutic window, i.e., 10–500 ng/ml in the current study.

Although regenerative medicine approaches for bone healing have relied heavily on bone marrow-derived MSCs since MSCs can be derived from a multitude of tissues, a likely possibility is that strategies for improving fracture healing bone repair will include the application of cells derived from tissues other than bone that may provide logistical, and potentially biological advantages. An obvious example is the use of adipose tissue, which from a logistical standpoint is practical due to its relative abundance and the potential to provide a large number of MSCs, in addition to the limited invasiveness and technical limitations for the procurement of ASCs (Zuk et al., 2001). Despite the potential benefits of using ASCs, their osteogenic capacity is still a matter of contention as a limitation to their application for bone repair (Hattori et al., 2006; Hayashi et al., 2008). In the current study the effect of BMPs-2, -4, -6, -7, or -9 on the osteogenic differentiation of ASCs was evaluated to determine the most appropriate BMP for the osteogenic differentiation of ASCs. Similar to that observed with C_2_C_12_ cells, there was an increase in the ability of several of the BMPs to increase ALP activity as compared to control, with BMP-9, and BMPs-6 and -9 being superior to all other BMPs at days 3 and 7, respectively (Figure 2). Although there was an almost 2-fold increase in ALP activity with the best doses as compared to control, the absolute ALP activity achieved was still ~10-fold less in ASCs as compared to C_2_C_12_ cells (Figure 1 vs. Figure 2). Nonetheless, the superior ability of BMPs-6 and -9, BMP-9 in particular, suggests these BMPs may be particularly useful for osteogenic differentiation when considering the need to differentiate cells that have varying levels of osteogenic potential.

In addition to the necessity for osteogenic differentiation, the process of fracture healing includes cell migration, cell proliferation, and angiogenesis, which involves a variety of regenerative cells at the site of fracture healing (Marsell and Einhorn, 2011). Of these regenerative cells, BMSCs are a particularly attractive target given their more recently characterized and increasingly appreciated ability to secrete factors that potentially influence healing through their interaction with other cells within the fracture environment (Guo et al., 2006; Geiger et al., 2007; Shinohara et al., 2011). To this end, growth factors with significant roles in cell recruitment (SDF-1), angiogenesis (VEGF), and angiogenesis and mitogenesis (b-FGF) were evaluated after BMP exposure. Collectively, although the various BMPs were capable of altering BMSC secretion as when compared to untreated controls, the emergence of a particular BMP as having a significant advantage was less apparent than that was observed for osteogenic differentiation. An obvious limitation to the current approach is that a single relatively early time point was chosen, however, it can be argued that the presence of these factors during the early phases of BMSC appearance at the site of fracture healing improves the environment to enable successful regeneration.

Of the factors secreted by hBMSCs during BMP exposure, a surprising outcome was found with the use of BMP-5, which was not particularly osteogenic (Figures 1, 2), in the secretion of SDF-1. SDF-1 is a chemokine that binds CXCR4 which influences chemotaxis of lymphocytic cells to areas of inflammation and homing of stem/progenitor cells during development and tissue regeneration (Libura et al., 2002). The current findings suggest that through SDF-1 up-regulation, BMP-5 could help modulate proper trafficking of immune cells and progenitor cells to an injured area. Once relevant cells have migrated to the wound environment, both the osteogenic differentiation of relevant cells and the initiation of angiogenesis, especially that which can be achieved with modified BMSCs, are two important processes that impact overall healing (Guo et al., 2006; Geiger et al., 2007). Both BMPs-4 and -5 were effective at improving VEGF secretion as compared to the other BMPs, the former being in agreement with previous reports that BMP-4 has a positive effect on angiogenesis (David et al., 2009). If angiogenic capacity is inferred from the ability to improve both b-FGF and VEGF secretion (Figures 3B,C), BMP-2 was superior to all others tested. In other words, BMP-2 was capable of improving both b-FGF and VEGF whereas other BMPs only improved VEGF. This somewhat correlates with clinical findings since high doses of BMP-2 was also associated with a reduced infection rate following Type III open tibia fractures, potentially because of its ability to augment vascular supply to the injured area (Govender et al., 2002). BMP-2 gene expression is up-regulated with in the first 24 h after fracture during the inflammation phase of healing (Cho et al., 2002; Ai-Aql et al., 2008; David et al., 2009). A logical speculation is that the production of both acid and basic FGF as a result of BMP-2 exposure during the early phases of healing, promotes the induction of bone marrow derived stem cells to become osteoblasts while also improving angiogenesis and mitogenesis (Einhorn et al., 2007).

Deviating from the therapeutic application of the various BMPs an attempt was made to gain more insight into the role of endogenous BMP levels of BMSCs while under osteogenic induction conditions. The endogenous expression of BMP-2, -4, and -6 with little constitutive expression of BMPs-7 and -9 have been previously reported (Seib et al., 2009). In agreement with these findings in the current study, the expression levels of BMP-7 and -9 expression levels were barely detectable (data not shown). Overall, this data should be interpreted with caution given the low sample number, however, the relatively small changes in gene expression of markers of osteogenesis and growth factors despite significant decreases in BMPs-2 and -6 suggest that significant redundancy exists within the cells' genetic machinery to respond to osteogenic cues. Decreases in the levels of BMP-2 or BMP-6 in hBMSCs by 52 and 94%, respectively, were insufficient to affect ALP or osteocalcin expression (Figures 4A,B). Given the ability of other BMPs, specifically BMP-9, to affect osteogenesis it is plausible that sufficient redundancy exists to compensate for the deficiency of either of these BMPs. The observation that BMP-6 knockdown caused an increase in BMP-2 expression (Figure 4B) provides evidence to support this speculation. Whether other BMPs were up-regulated to compensate for a deficiency in BMP-2 or -6 was not determined. Nonetheless, decreases in osteoblast BMP-2 and BMP-6 mRNA caused decrements (although not significant) in ALP and osteocalcin expression. Differences in ALP and osteocalcin between hBMSCs and osteoblasts at this early time point may be reflective of osteoblasts being further along the differentiation process. The lack of change in b-FGF expression with BMP-2 knockdown is interesting considering the ability of exogenous BMP-2 to improve its secretion, however, cell signaling cascades that affect growth factor production with exogenous exposure of BMPs are likely affected differently when endogenous expression of genes is manipulated. With knockdown of both BMP-2 and -6 in hBMSCs and osteoblasts, VEGF mRNA expression increased. This was an interesting observation. Our postulation for the reason VEGF increased in the absence of BMPs is that the cells were possibly attempting to compensate for the decrease in BMPs as it has been shown that VEGF can increase BMP-2 mRNA (Bouletreau et al., 2002). Furthermore, given that VEGF secretion was decreased at the highest level of BMP-6 used (Figure 3B), it was interesting to observe a significant increase in VEGF gene expression with BMP-6 knockdown. Whether BMP-6 plays a unique role in the regulation of VEGF production or *vice versa* requires further investigation.

In summary, the findings herein add to the growing body of literature that suggests there are the divergent effects among the various BMPs, especially those other than the clinically used BMPs-2 and -7. When deciding which of the BMPs is the most appropriate for improving bone regeneration other processes related to bone regeneration including, but not limited to, the effect on the secretory capacity of growth factors involved in repair needs to be considered. Bone healing is a carefully orchestrated event that must have appropriate biological cues to affect regenerative events at the right time. In this regard, our observations support the idea that there is a divergence among the BMPs and the processes that they influence. In light of this idea, it is logical to propose that temporal administration of several BMPs be used to maximize bone repair. Future investigations are also needed to delineate how multiply delivered BMPs can augment the combined processes required for fracture healing. In summary, these findings support efforts to study other BMPs as potential bone graft supplements, and to consider combined BMP delivery for promotion of multiple aspects of fracture healing.

## Author contributions

Jessica C. Rivera, Joseph C. Wenke, and Christopher R. Rathbone performed study design of all *in vitro* work. Jessica C. Rivera and Christopher R. Rathbone conducted laboratory work for all *in vitro* work. Cassandra A. Strohbach performed design and execution of PCR work. Jessica C. Rivera and Christopher R. Rathbone performed data analysis and production of the above manuscript. Joseph C. Wenke and Cassandra A. Strohbach provided significant editorial assistance with production of the above manuscript.

### Conflict of interest statement

The authors declare that the research was conducted in the absence of any commercial or financial relationships that could be construed as a potential conflict of interest.
